# Influencing Factor Investigation on Dynamic Hydrothermal Growth of Gapped Hollow BaTiO_3_ Nanospheres

**DOI:** 10.1186/s11671-015-1033-x

**Published:** 2015-08-18

**Authors:** Jiabing Gao, Haiyue Shi, Jing Yang, Tao Li, Rui Zhang, Deliang Chen

**Affiliations:** School of Materials Science and Engineering, Zhengzhou University, 100 Science Road, Zhengzhou, 450001 People’s Republic of China; Laboratory of Aeronautical Composites, Zhengzhou Institute of Aeronautical Industry Management, University Centre, Zhengdong New District, Zhengzhou, 450046 People’s Republic of China

**Keywords:** Crystal morphology, Hydrothermal crystal growth, Nanomaterials, BaTiO_3_ nanosphere

## Abstract

Gapped hollow BaTiO_3_ nanospheres with an apparent diameter of 93 ± 19 nm (shell thickness of 10–20 nm) were synthesized via a dynamic hydrothermal process using TiO_2_ sols and Ba^2+^ ions as the Ti and Ba sources in alkaline aqueous solutions. The phases and morphologies of the BaTiO_3_ samples were characterized by X-ray diffraction (XRD), SEM, TEM, and Raman spectra. The effects of the hydrothermal temperatures and durations, NaOH concentrations, and Ba/Ti ratios on the formation of gapped hollow BaTiO_3_ nanospheres were systematically investigated. The optimum conditions for forming gapped hollow BaTiO_3_ nanospheres are hydrothermal treatment at 180 °C for 10–20 h under a continuous magnetic stirring with NaOH concentrations of about 1 mol/L and molar Ba/Ti ratios of 1.2–1.5. The formation mechanism of the gapped hollow BaTiO_3_ nanospheres is understood as the combination of the orientated attachment and reversed crystal growth.

## Background

Hollow nanostructures with unique microstructures and functional properties have attracted increasing attention in scientific and engineering aspects [1, 2]. Hollow nanostructures show enhanced functional properties because of their high surface areas [3, 4]. The Kirkendall effect, Ostwald ripening, and hard/soft templates have also been used to construct hollow nanostructures [5–7]. However, the fine control on the formation of hollow nanostructures and deep understanding of the related mechanisms are still a big challenge.

Barium titanate (BaTiO_3_, BTO), a high-k dielectric material, has been widely applied in commercial multilayer ceramic capacitors [8, 9]. Being a lead-free ferroelectric ceramic, BTO is an environment-friendly material for various applications, including capacitors [10], electronic devices [11, 12], ultrasonic transducer [13], energy storage capacity [14], microwave absorbers [15, 16], and semiconductors [17–19]. Barium titanate is usually of four different phases which depend on the formation temperature: the paraelectric cubic, ferroelectric tetragonal, orthorhombic, and rhombohedral phases [19]. Hollow structures can improve the dielectric properties of BaTiO_3_ nanoparticles [20, 21]. Some methods have been developed in the past decades to fabricate BaTiO_3_ hollow nanostructures, such as the layer-by-layer colloidal templating method [21], molten hydrated salt method [22], and doping method [23]. These methods for the synthesis of hollow BaTiO_3_ nanostructures are complicated, and simpler processes are required.

Wet chemical methods have widely been developed to synthesize nanoscale powders (5–100 nm) with advantages of high purity, near-atomic level homogeneity, and adjustable compositions [24–26]. Among the wet chemical methods, the hydrothermal process is thought as a versatile, low-cost and environment-friendly method to prepare barium titanate nanoparticles with controlled sizes and morphologies [20, 27, 28]. For example, Geng et al. [29] developed a general hydrothermal process using stick-like titania powders, sodium hydroxide, and soluble target ions as the starting materials to achieve MTiO_3_ (M = Sr, Ba, and Ca) polyhedra. Hollow (bowl-like) BaTiO_3_ particles can also be synthesized via a hydrothermal process [30]. Although there are reports on the hydrothermal synthesis of barium titanate nanocrystals [20, 28, 31], the influencing factors and formation mechanism of the barium titanate nanocrystals (especially the hollow nanospheres) are still not clear. It is necessary to investigate the fundamental roles of the synthetic parameters (temperatures, precursors, times, etc.) on the crystallization thermodynamics and kinetics of anisotropic growth of barium titanate [26, 32]. Hydrothermal syntheses of BaTiO_3_ particles with the aid of surfactants (i.e., PEG) are well investigated [28, 33], but these syntheses of hollow BaTiO_3_ particles without surfactants are seldom reported [30].

**Scheme 1:**
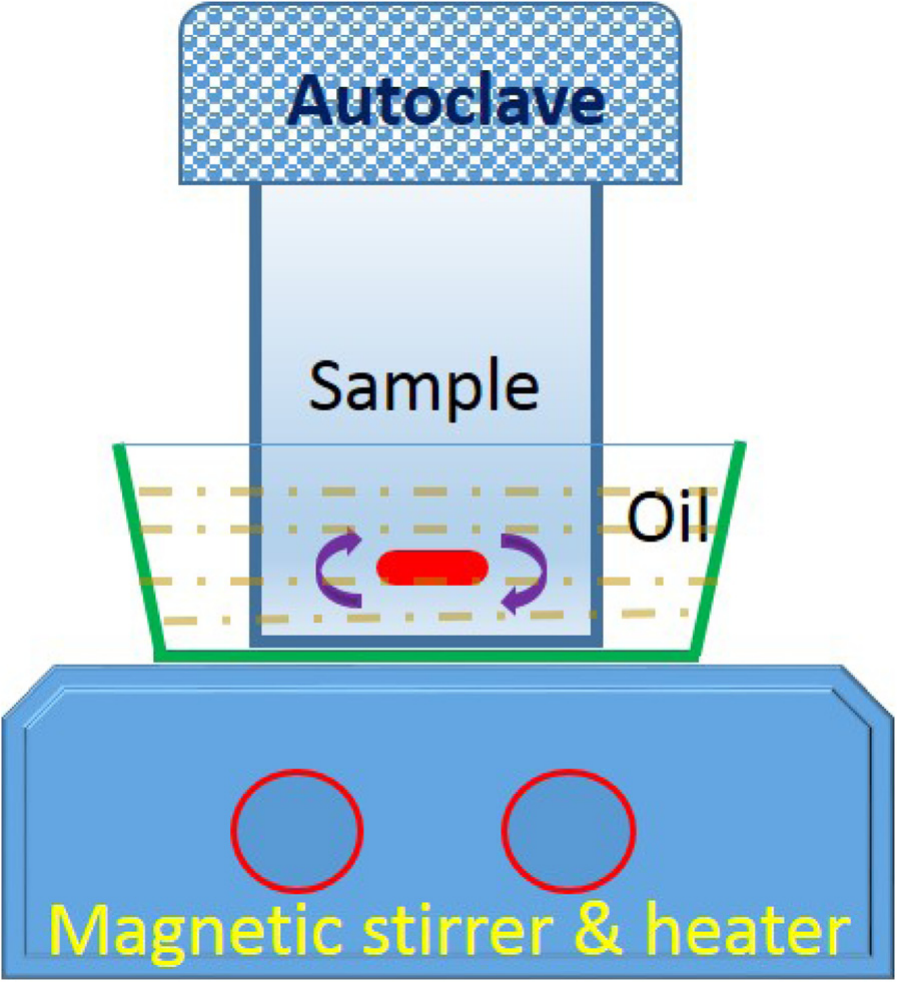
The setup for dynamic hydrothermal synthesis of gapped hollow BaTiO_3_ nanospheres

In this work, we developed a dynamic hydrothermal process to synthesize gapped hollow BaTiO_3_ nanospheres without surfactants, just by modifying the common static hydrothermal process [34] with a continuous magnetic stirring during the whole crystallization process (Scheme 1). The effects of hydrothermal temperatures, the molar Ba/Ti ratios, NaOH concentrations, and hydrothermal durations on the formation of gapped hollow BaTiO_3_ nanospheres are systematically investigated. The morphologies, particle sizes, and phase compositions of the as-obtained BaTiO_3_ particles are carefully characterized. The relationships between the phases, microstructures, and synthetic conditions are investigated.

## Methods

Tetrabutyl titanate (Ti(OC_4_H_9_)_4_, analytically pure) and barium nitrate (Ba(NO_3_)_2_, chemically pure) were purchased from Sinopharm Chemical Reagent Co., Ltd.; sodium hydrate (NaOH, analytically pure), glacial acetic acid (C_2_H_4_O_2_, analytically pure), and isopropyl alcohol (C_3_H_8_O, analytically pure) were purchased from Tianjing Kermel Chemical Reagent Co., Ltd.; and ethanol (analytically pure) was purchased from Anhui Ante Biochemical Co., Ltd. All reagents were used as received without further purification. Distilled water was used in the experiments.

Gapped hollow BaTiO_3_ nanospheres were synthesized by treating the mixtures of TiO_2_ sols and Ba(NO_3_)_2_ under a dynamic hydrothermal condition (Scheme 1). For the preparation of TiO_2_ sols, Ti(OC_4_H_9_)_4_ (80 mL) was mixed with isopropyl alcohol (80 mL) to form a homogeneous solution, which was then added to an acidic solution made by mixing glacial acetic acid (30 mL) and distilled water (800 mL). The above suspension was kept stirring for 3–5 days at room temperature to form a semitransparent TiO_2_ sol. For the synthesis of gapped hollow BaTiO_3_ nanospheres, Ba(NO_3_)_2_ solids were firstly dissolved to 60 mL of TiO_2_ sol, and the as-obtained sol was then mixed with a NaOH solution (20 mL). During the above mixtures, the molar Ba/Ti ratios (*R*_Ba/Ti_) were kept 1–2, and the NaOH concentrations ([NaOH]) were 1–3 mol/L. The suspension (~80 mL) was then transferred to a 100-mL Teflon-lined stainless steel autoclave with a magnetic stirrer and tightly sealed. Then, the autoclave was placed in oil bath (100–200 °C) under a magnetically stirring condition for 30 min to 50 h. After reaction for given times, the solid samples were collected and washed several times, followed by drying at 120 °C for about 12 h. The detailed experimental conditions for the synthesis of BaTiO_3_ samples are summarized in Table 1.

**Table 1:** A summary of experimental conditions for the synthesis of BaTiO_3_ nanocrystals via the dynamic hydrothermal process

Sample	[NaOH] (mol/L)	*R*_Ba/Ti_	Hydrothermal temperature (°C)	Hydrothermal duration (h)
S1	1.0	1.2	180	20
S2	1.5	1.2	180	10
S3	2.0	1.2	180	10
S4	2.5	1.2	180	10
S5	3.0	1.2	180	10
S6	1.0	1.0	180	10
S7	1.0	1.2	180	10
S8	1.0	1.5	180	10
S9	1.0	2.0	180	10
S10	1.0	1.2	100	10
S11	1.0	1.2	150	10
S12	1.0	1.2	200	10
S13	1.0	1.2	180	0.5
S14	1.0	1.2	180	1
S15	1.0	1.2	180	3
S16	1.0	1.2	180	5
S17	1.0	1.2	180	30
S18	1.0	1.2	180	1/6
S19	1.0	1.2	180	40
S20	1.0	1.2	180	50

The phase compositions of the as-obtained samples were determined using X-ray diffraction (XRD) performed on an XD-3 X-ray diffractometer (Beijing Purkinje General Instrument Co., Ltd., China) with Cu Kα irradiation (*λ* = 0.15406 nm). The morphologies and microstructures of the samples were observed using a field-emission electron scanning microscope (FE-SEM; JEOL 7500F) and a field-emission transmission electron microscope (FE-TEM; Tecnai G^2^ F20, accelerating voltage of 200 kV, Philips) with an attachment of energy-dispersive analysis of X-ray (EDAX). Raman spectra were recorded in the 100–1000 cm^−1^ wavenumber range using a Horiba Xplora Raman microscope (Horiba Scientific).

## Results and Discussion

### Phases, Compositions, and Morphologies of Gapped Hollow BaTiO_3_ Nanospheres

**Fig. 1:**
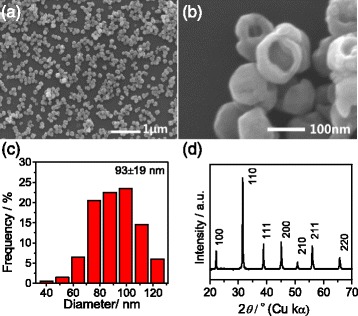
**a**, **b** Typical SEM images, **c** size-distribution histogram, and **d** XRD pattern of the gapped hollow BaTiO_3_ nanospheres (S1) obtained via dynamically hydrothermal treatment at 180 °C for 20 h with [NaOH] = 1.0 mol/L and *R*_Ba/Ti_ = 1.2

Gapped hollow BaTiO_3_ nanospheres have been synthesized via a simple dynamic hydrothermal process. Figure 1 shows the SEM observations and XRD pattern of the typical BaTiO_3_ sample (S1 in Table 1) obtained at 180 °C for 20 h with [NaOH] = 1 mol/L and *R*_Ba/Ti_ = 1.2. Figure 1a shows a low-magnification SEM image, which suggests that the as-obtained sample consists of well-dispersed nanospheres with uniform morphology and size. The enlarged SEM image in Fig. 1b shows that the sphere-like nanoparticles are of a hollow and gapped microstructure. The apparent particle sizes of the gapped hollow BaTiO_3_ spheres are 93 ± 19 nm according to the statistical analysis of SEM observations, as shown in Fig. 1c. The shell thickness of the hollow sphere is about 10–20 nm according to the SEM observation (Fig. 1b). The phase composition of the gapped hollow BaTiO_3_ nanospheres is determined by XRD measurement, and a typical XRD pattern is shown in Fig. 1d. The sample can be indexed to a mixture of cubic and tetragonal BaTiO_3_ phases [11, 33]. Actually, it is difficult to distinguish the tetragonal BaTiO_3_ phase from the cubic one just by XRD patterns. If the as-obtained BaTiO_3_ sample is thought as a cubic BaTiO_3_ phase (space group, Pm3, *a* = 4.03 Å, JCPDS card no. 31-0174), the obvious peaks (2*θ*) at around 22.12°, 31.52°, 38.81°, 45.17°, 50.80°, 56.10°, and 65.76° belong to the (100), (110), (111), (200), (210), (211), and (220) reflections of cubic BaTiO_3_ phase, respectively. The calculated cell parameter is *a* = 4.0135(8) Å using a UnitCell program (a method developed by TJB Holland and SAT Redfern) by minimizing the sum of squares of residuals in 2*θ*. The XRD pattern of S1 is similar to that of the literature result of ref. [33], which is indexed to a cubic BaTiO_3_ phase. Therefore, it seems safe that the S1 sample mainly consists of cubic BaTiO_3_ phase.

**Fig. 2:**
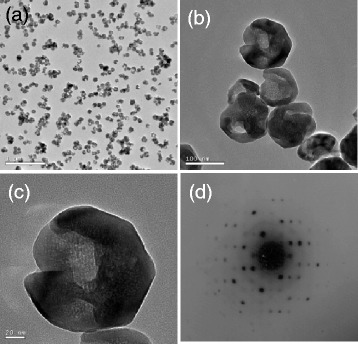
TEM observations of the gapped hollow BaTiO_3_ nanospheres (S1) obtained via dynamically hydrothermal treatment at 180 °C for 20 h with [NaOH] = 1.0 mol/L and *R*_Ba/Ti_ = 1.2, **a**−**c** TEM images with various magnifications, and **d** SAED pattern

To confirm the hollow and gapped spherical morphology, we observed the as-obtained BaTiO_3_ sample using a TEM microscope, and the typical results are shown in Fig. 2. The low-magnification TEM image in Fig. 2a shows that the BaTiO_3_ particles are uniform, highly dispersed, and semitransparent, seeming of a hollow structure. The high-magnification TEM image in Fig. 2b definitely shows that the BaTiO_3_ particles are of a hollow and gapped spherical morphology with an apparent diameter of ~100 nm. The thickness of the gapped hollow sphere in Fig. 2c is less than 20 nm. Figure 2d shows the corresponding selected area electron diffraction (SAED) pattern of the BaTiO_3_ particle (Fig. 2c). One can see that the SAED pattern consists of several sets of diffraction spots, indicating that the gapped hollow BaTiO_3_ particle is composed of several parts and each of them is of a local single-crystalline structure. The results of TEM observations well agree with the SEM results in Fig. 1. Dai et al. [30] have reported a successful synthesis of bowl-like single-crystalline BaTiO_3_ nanoparticles via a conventional hydrothermal process using Ba(OH)_2_·8H_2_O and TiO_2_ as precursors. The gapped hollow BaTiO_3_ nanospheres obtained in the present synthesis are similar to the above bowl-like nanoparticles in morphology although the synthetic process is very much different from each other.

**Fig. 3:**
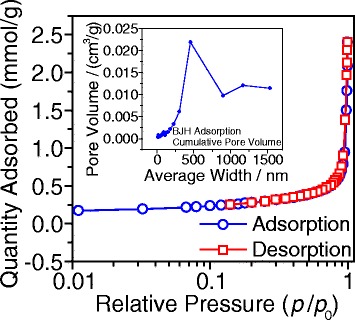
Nitrogen adsorption-desorption isotherm curves and the pore-size distribution based on the BJH method (the *ins*) of the gapped hollow BaTiO_3_ nanospheres (S1) obtained via dynamically hydrothermal treatment at 180 °C for 20 h with [NaOH] = 1.0 mol/L and *R*_Ba/Ti_ = 1.2

The microstructure of the gapped hollow BaTiO_3_ nanospheres is also characterized by N_2_ adsorption-desorption test. Figure 3 shows the typical N_2_ adsorption-desorption isotherms and pore-size distribution curve. The adsorption isotherm shows an obvious increase at higher relative pressure, indicating that there is a weak interaction between N_2_ molecules and gapped hollow BaTiO_3_ nanospheres. Similarly, the desorption isotherm in Fig. 3 shows a sharp decrease with the decrease in the relative pressure. It is noteworthy that the absorption curve does not overlap with the desorption one, suggesting that the N_2_ molecules are not totally reversible at desorption. The pore-size distribution curve is shown as the inset of Fig. 3, which indicates that there are some large pores with sizes of 250–750 nm. Those large pores probably result from the loose aggregation of the gapped hollow BaTiO_3_ nanospheres (Figs. 1 and 2). The specific Brunauer-Emmett-Teller (BET) surface area is calculated to be 22.5 m^2^/g.

### Factors Influencing the Formation of Gapped and Hollow BaTiO_3_ Nanospheres

**Fig. 4:**
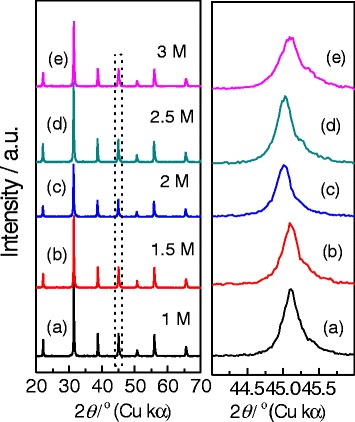
Typical XRD patterns at around 2*θ* = 20°–70° and 2*θ* = 44°–46° of the BaTiO_3_ samples synthesized via a dynamic hydrothermal process at 180 °C for 10 h (*R*_Ba/Ti_ = 1.2) with various NaOH concentrations: **a** 1.0 mol/L (S2), **b** 1.5 mol/L (S3), **c** 2.0 mol/L (S4), **d** 2.5 mol/L (S5), and **e** 3.0 mol/L (S6)

We firstly investigate the effects of NaOH concentrations on the phase composition and morphology of BaTiO_3_ nanocrystals derived via the dynamic hydrothermal process. From the thermodynamic point of view, BaTiO_3_ can be easily formed in strong alkaline conditions. To check the effect of NaOH concentrations on the crystallinity and morphology, we change the NaOH concentrations in a range of 1–3 mol/L to synthesize BaTiO_3_ samples. Figure 4 shows the typical XRD patterns of the BaTiO_3_ samples synthesized with various NaOH concentrations at 180 °C for 10 h with *R*_Ba/Ti_ = 1.2. As the XRD patterns in the 2*θ* range of 20°–70° show (left in Fig. 4), the as-synthesized samples show similar XRD patterns, which can be indexed to BaTiO_3_ phases (tetragonal (JCPDF no. 05-0626) or cubic (JCPDF no. 31-0174)), and no impure phases can be found [11, 33, 35]. The enlarged XRD patterns in the 2*θ* range of 44°–46° show that the XRD peak at around 45° becomes wider and wider as the NaOH concentration increases from 1 to 3 mol/L, indicating that the higher NaOH concentration is favorable in forming tetragonal BaTiO_3_ phase [26, 35].

**Fig. 5:**
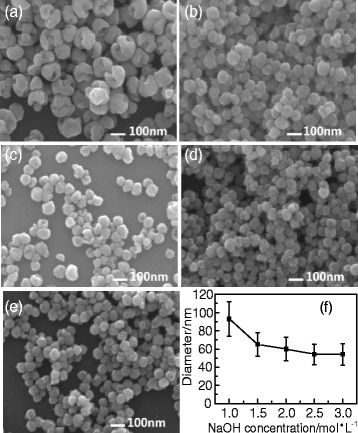
**a**−**e** SEM images of the BaTiO_3_ samples synthesized via a dynamic hydrothermal process at 180 °C for 10 h (*R*_Ba/Ti_ = 1.2) with various NaOH concentrations: **a** 1.0 mol/L (S2), **b** 1.5 mol/L (S3), **c** 2.0 mol/L (S4), **d** 2.5 mol/L (S5), and **e** 3.0 mol/L (S6). **f** The change of the particle sizes of the BaTiO_3_ samples as function of NaOH concentrations

Figure 5 shows the typical SEM observations of the BaTiO_3_ samples synthesized with different NaOH concentrations via the dynamic hydrothermal process at 180 °C for 10 h with a Ba/Ti ratio of 1.2. As the SEM images shown in Fig. 5a–e, one can see that low NaOH concentrations (e.g., [NaOH] = 1 mol/L in Fig. 5a) are favorable in forming hollow BaTiO_3_ nanospheres, whereas the high concentrations (e.g., [NaOH] = 3 mol/L in Fig. 5e) improve the formation of solid BaTiO_3_ nanospheres. Also, the NaOH concentrations have some effects on the particle sizes of the as-obtained BaTiO_3_ samples. Figure 5f shows the plot of the particle sizes as function of NaOH concentrations, and their particle sizes were statistically analyzed on the basis of SEM images. One can see that the particle sizes of the as-obtained BaTiO_3_ samples decrease from about 90 to 60 nm as the NaOH concentrations increase from 1 to 3 mol/L. At the same time, the gapped and hollow structures disappear and a solid spherical nanostructure is formed. One can think that the amount of hydroxyl groups increases as the NaOH concentration increases. In a high [NaOH] solution, the nuclei have more hydroxyl groups on their surfaces, which favor the growth of negatively charged BaTiO_3_ nanoparticles at a pH ≥10 solution. The negatively charged BaTiO_3_ nanoparticles can repel each other because of the electrostatic repulsion, which prevents from agglomerating and enhance the dispersibility of the BaTiO_3_ nanoparticles.

**Fig. 6:**
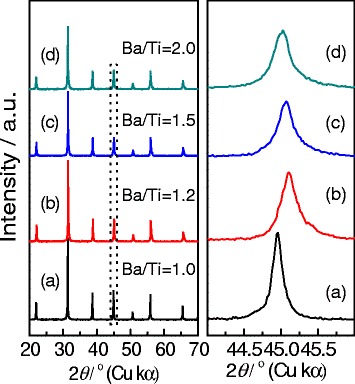
Typical XRD patterns at around 2*θ* = 20°–70° and 2*θ* = 44°–46° of the BaTiO_3_ samples synthesized via the dynamic hydrothermal process at 180 °C for 10 h ([NaOH] = 1.0 mol/L) with various Ba/Ti ratios: **a** 1.0 (S6), **b** 1.2 (S7), **c** 1.5 (S8), and **d** 2.0 (S9)

We then checked the effects of the Ba/Ti ratios on the formation of gapped hollow BaTiO_3_ nanospheres in phase compositions and morphologies. We synthesized BaTiO_3_ samples at 180 °C for 10 h with various Ba/Ti ratios (*R*_Ba/Ti_ = 1.0–2.0) via the dynamic hydrothermal process in aqueous solutions with [NaOH] = 1 mol/L. Figure 6 shows the XRD patterns of the as-obtained BaTiO_3_ samples. One can see that the XRD patterns show similar peaks that can be indexed to pure BaTiO_3_ phase [11, 12]. The enlarged XRD peaks at around 45 °C show that the peaks belonging to {200} deflections become wider and wider as the *R*_Ba/Ti_ increases from 1 to 2, suggesting that higher *R*_Ba/Ti_ values favor the formation of tetragonal BaTiO_3_ phase [11, 35].

**Fig. 7:**
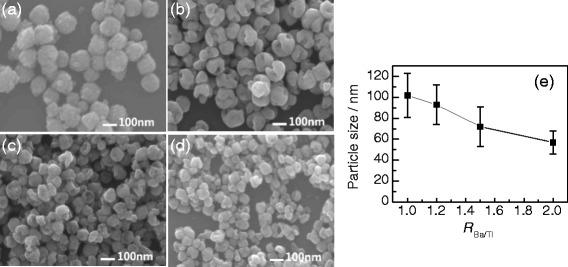
**a**–**d** SEM images of BaTiO_3_ samples synthesized via the dynamic hydrothermal process at 180 °C for 10 h with [NaOH] = 1.0 mol/L and different Ba/Ti ratios: **a** 1.0 (S6), **b** 1.2 (S7), **c** 1.5 (S8), and **d** 2.0 (S9). **e** The plot of the particle sizes of the BaTiO_3_ samples as a function of Ba/Ti ratios

The morphologies of the BaTiO_3_ samples obtained with various Ba/Ti ratios (*R*_Ba/Ti_ = 1.0–2.0) were observed using a SEM microscope. Figure 7 shows the typical SEM images and the corresponding particle-size distribution plot. Figure 7a shows the SEM image of the BaTiO_3_ sample obtained with *R*_Ba/Ti_ = 1, and the sample takes on a spherical morphology. The BaTiO_3_ spheres are of a size range of 102 ± 21 nm and formed by aggregating small nanoparticles. When the *R*_Ba/Ti_ increases to 1.2, gapped hollow BaTiO_3_ nanospheres are obtained, as shown in Fig. 7b. As the *R*_Ba/Ti_ value continues to increase to 1.5 and 2.0, the BaTiO_3_ samples obtained show definitely hollow spherical morphologies (Fig. 7c, d). Figure 7e gives the particle-size change of the BaTiO_3_ samples with the *R*_Ba/Ti_ values. One can see that the sizes decrease from 102 ± 21 to 57 ± 11 nm as the *R*_Ba/Ti_ value increases from 1 to 2. The XRD (Fig. 6) and SEM (Fig. 7) results indicate that higher *R*_Ba/Ti_ values favor the formation of gapped hollow BaTiO_3_ nanospheres with smaller sizes and more amount of tetragonal phase [22].

**Fig. 8:**
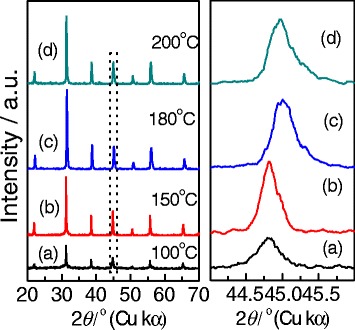
Typical XRD patterns at around 2*θ* = 20°–70° and 2*θ* = 44°–46° of the BaTiO_3_ samples synthesized via the dynamic hydrothermal process with *R*_Ba/Ti_ = 1.2 and [NaOH] = 1.0 mol/L at various temperatures for 10 h: **a** 100 °C (S10), **b** 150 °C (S11), **c** 180 °C (S7), and **d** 200 °C (S12)

**Fig. 9:**
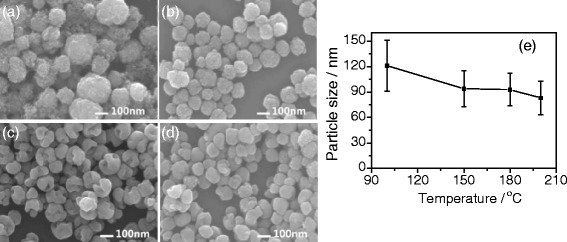
**a**-**d** SEM images of BaTiO_3_ samples synthesized via the dynamic hydrothermal process with *R*_Ba/Ti_ = 1.2 and [NaOH] = 1.0 mol/L at various temperatures for 10 h: **a** 100 °C (S10), **b** 150 °C (S11), **c** 180 °C (S7), and **d** 200 °C (S12). **e** The plot of the particle sizes of the BaTiO_3_ samples obtained at various temperatures

We finally investigated the effects of hydrothermal temperatures on the formation of gapped and hollow BaTiO_3_ nanospheres during the dynamic hydrothermal synthesis. The experiments were done under the same Ba/Ti ratio (*R*_Ba/Ti_ = 1.2), NaOH concentration ([NaOH] = 1.0 mol/L), and hydrothermal time (10 h), and the hydrothermal temperatures were varied from 100 to 200 °C. Figures 8 and 9 show the typical XRD patterns and SEM observations of the as-obtained BaTiO_3_ samples. As Fig. 8 shows, all the samples can be indexed to cubic (JCPDF no. 31-0174) or tetragonal (JCPDF no. 05-0626) BaTiO_3_ phases [12, 36]. The intensity and sharpness of the diffraction peaks increased with the reaction temperatures, indicating a continuous increase of the crystallinity. The widened XRD peaks at around 2*θ* of 45° possibly from (200) and (002) reflections for the samples obtained at higher temperatures (e.g., 180 and 200 °C) indicate that the higher hydrothermal temperatures are favorable in increasing the amount of tetragonal BaTiO_3_ phase.

Figure 9 shows the SEM images (a–d) and particle-size distribution (e) of the BaTiO_3_ samples synthesized at different hydrothermal temperatures. One can see that the BaTiO_3_ samples obtained at less than 150 °C consist of spheres formed by aggregating small particles, whereas the sample obtained at 200 °C mostly consists of solid spherical particles. As Fig. 9c shows, the samples obtained at 180 °C show a hollow and gapped spherical morphology. The particle-size plot in Fig. 9e shows that the particle sizes of the samples slightly decrease from 121 ± 30 to 83 ± 20 nm when the hydrothermal temperature increases from 100 to 200 °C.

Taking Figs. 4, 5, 6, 7, 8, and 9 into account, the optimum conditions for the formation of gapped hollow BaTiO_3_ nanospheres via the present dynamic hydrothermal process are at 180 °C, *R*_Ba/Ti_ = 1.2−1.5, and [NaOH] = 1.0 mol/L.

### Understanding of Growing Gapped Hollow BaTiO_3_ Nanospheres in Dynamic Hydrothermal Process

**Fig. 10:**
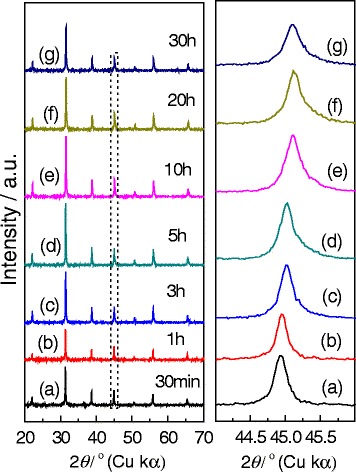
Typical XRD patterns at around 2*θ* = 20°–70° and 2*θ* = 44°–46° of the BaTiO_3_ samples synthesized via the dynamic hydrothermal process with *R*_Ba/Ti_ = 1.2 and [NaOH] = 1.0 mol/L at 180 °C for various times: **a** 30 min (S13), **b** 1 h (S14), **c** 3 h (S15), **d** 5 h (S16), **e** 10 h (S7), **f** 20 h (S1), and **g** 30 h (S17)

To investigate the growth process of gapped hollow BaTiO_3_ nanospheres, we synthesized a series of BaTiO_3_ samples with hydrothermal durations varying from 30 min to 30 h and the other conditions were kept the same: *R*_Ba/Ti_ = 1.2 and [NaOH] = 1.0 mol/L, at 180 °C for 10 h. Figure 10 shows the XRD patterns of the samples obtained. As the XRD patterns in the left column of Fig. 10 show, all the samples with various hydrothermal durations seem to consist of the same BaTiO_3_ phases [20, 23]. The enlarged XRD pattern at around 2*θ* = 45° (the right column in Fig. 10) shows that the peak positions did not experience a various shift from lower angles to higher ones as the hydrothermal durations increase from 30 min to 30 h, indicating the structure of the samples obtained is close to the cubic BaTiO_3_ phase [35]. However, the full width at half maximum (FWHM) values of the XRD patterns at around 2*θ* = 45° becomes wider and wider; this is due to the fact that these peaks are divided into (002) and (200) peaks. The (200) peak becomes more intense, its 2*θ* value is higher than the (002) one.

The position shifting and widening in FWHM of the XRD peaks at around 2*θ* = 45° suggests that the percentage of the tetragonal phase in the BaTiO_3_ sample increases with the increase in hydrothermal duration.

**Fig. 11:**
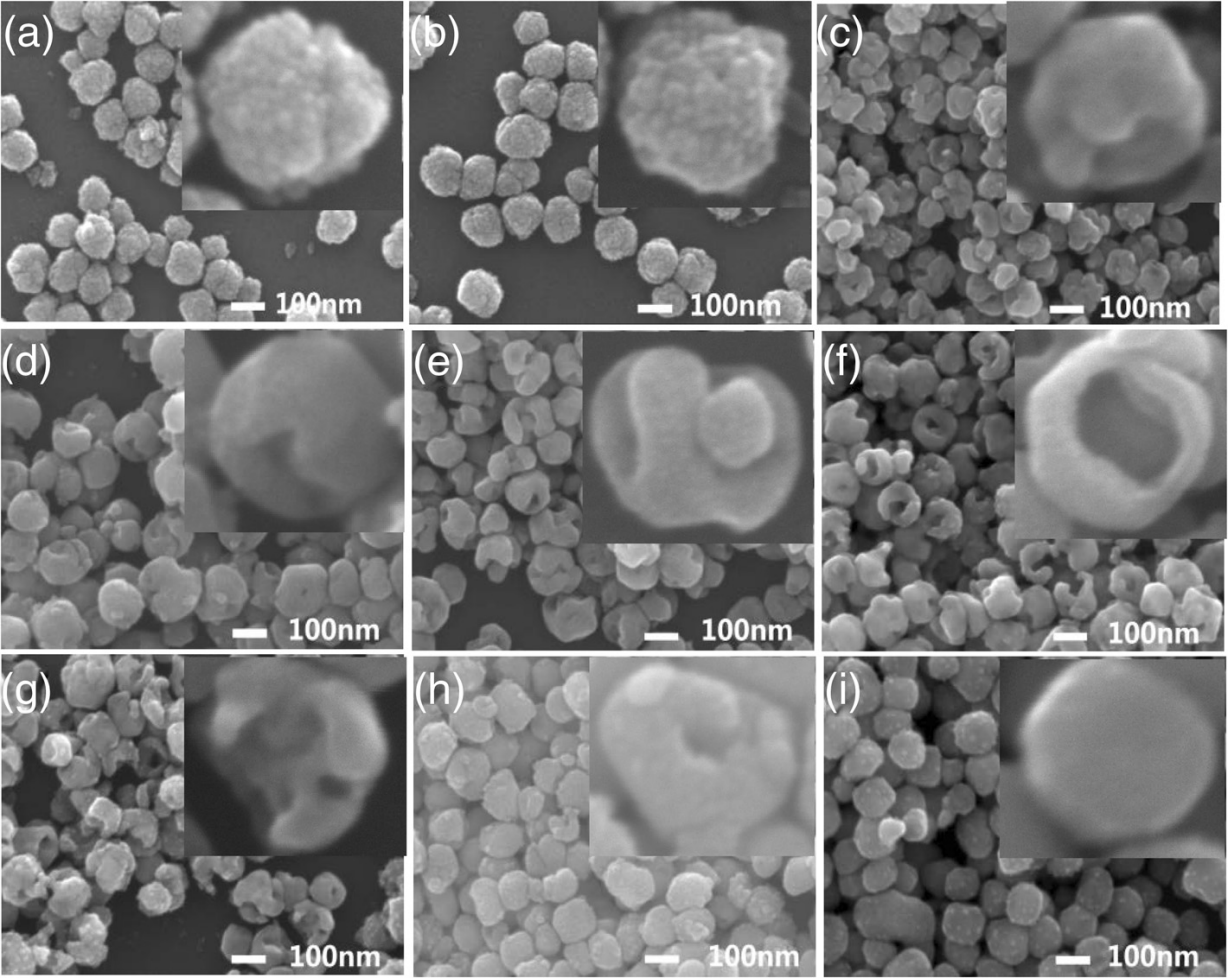
SEM images of BaTiO_3_ samples synthesized via the dynamic hydrothermal process with *R*_Ba/Ti_ = 1.2 and [NaOH] = 1.0 mol/L at 180 °C for various times: **a** 10 min (S18), **b** 1 h (S14), **c** 3 h (S15), **d** 5 h (S16), **e** 10 h (S7), **f** 20 h (S1), **g** 30 h (S17), **h** 40 h (S19), and **i** 50 h (S20)

Figure 11 shows the typical SEM images of the BaTiO_3_ samples obtained with various hydrothermal durations (form 10 min to 50 h). At the initial stages, i.e., Fig. 11a, b, the samples consist of solid spheres with about 100 nm in diameter, and the solid spheres are formed by aggregating smaller nanoparticles (5–10 nm). When the hydrothermal times are more than 3 h (e.g., 3–20 h), the BaTiO_3_ samples obtained tend to form gapped and hollow nanospheres, as shown in Fig. 11c−g. But when the hydrothermal duration is too long, for example, more than 30 h, the BaTiO_3_ samples tend to form solid nanospheres.

**Fig. 12:**
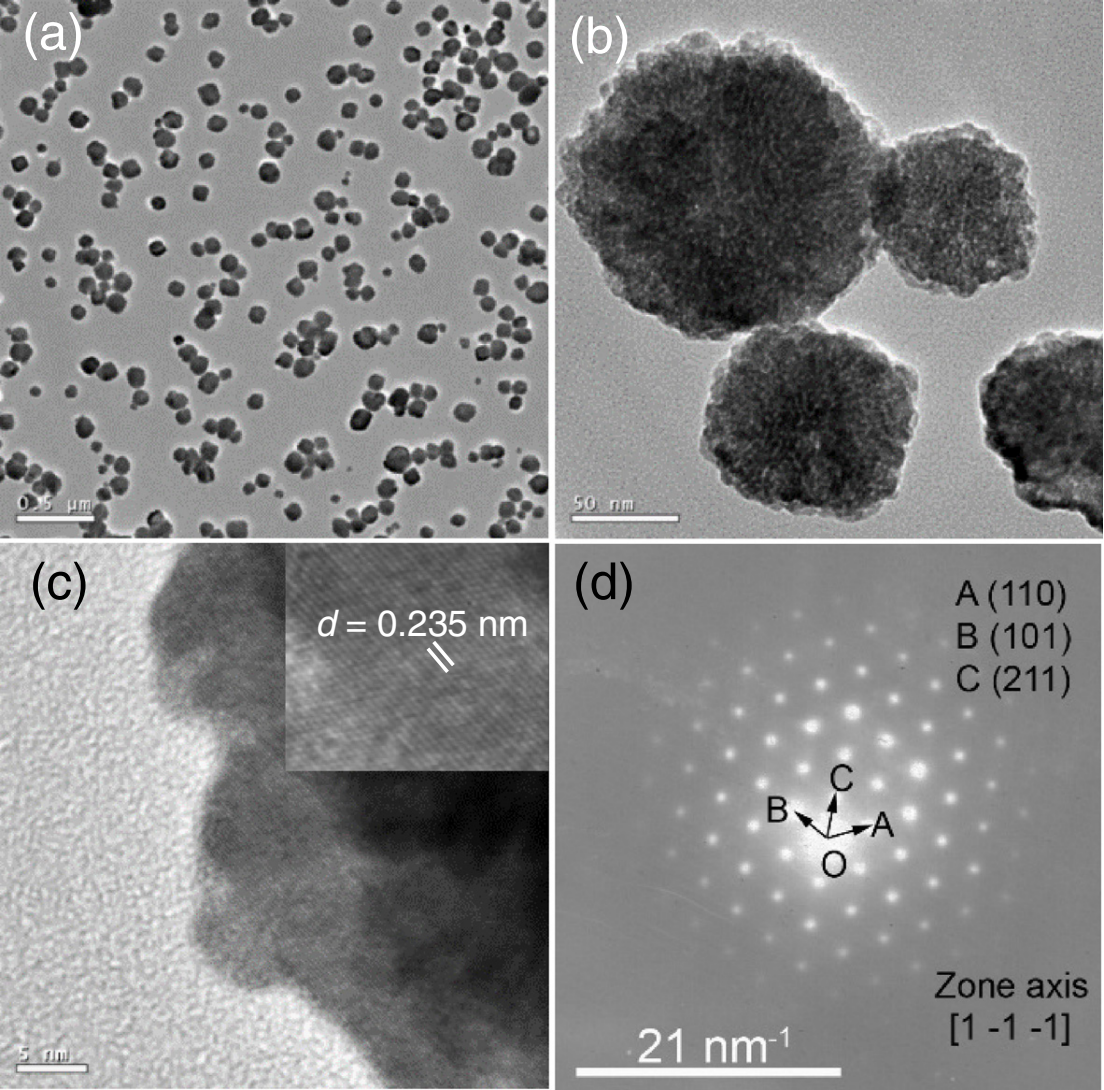
TEM observations of the BaTiO_3_ sample (S14) obtained via the dynamic hydrothermal process at 180 °C for 1 h with [NaOH] = 1.0 mol/L and *R*_Ba/Ti_ = 1.2: **a**-**b** low-magnification TEM images, **c** high-resolution TEM image, and **d** SAED pattern

To understand the growth mechanism, we observed a typical BaTiO_3_ sample obtained at the initial stage with a hydrothermal time of 1 h using the TEM technique. Figure 12 shows the typical TEM observations. The low-magnification TEM image in Fig. 12a shows that the as-obtained BaTiO_3_ sample with a short hydrothermal duration of 1 h is mainly composed of highly dispersed spherical particles with sizes of 80–120 nm. The enlarged TEM image shown in Fig. 12b indicates that the spherical particles are formed by loosely aggregating small nanoparticles (5–10 nm in size). Figure 12c shows the typical high-resolution TEM image, and the discernible lattice fringe of 0.235 nm (the inset of Fig. 12c) can be indexed to the interplanar distance of {111} planes of the cubic BaTiO_3_ phase (JCPDS no. 31-0174). Figure 12d shows the corresponding selected area electron diffraction (SAED) pattern, and this ordered pattern can be indexed to single-crystalline cubic BaTiO_3_ along the zone axis of [1 −1 −1]. The high-resolution TEM image and the corresponding SAED pattern indicate that the spherical particles are of a single-crystalline-like microstructure, suggesting that the nanoparticles are aggregated via an oriented attachment mechanism to form larger nanospheres at the initial stage during the dynamic hydrothermal process [37].

**Fig. 13:**
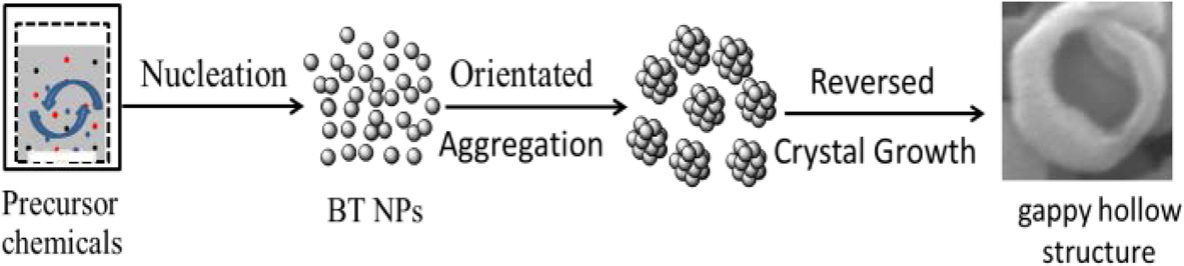
Formation paths of the gapped hollow BaTiO_3_ nanospheres in the dynamic hydrothermal process

Taking the XRD (Fig. 10), SEM images (Fig. 11), and TEM observations (Fig. 12) into account, we can safely conclude the possible growth paths of the gapped hollow BaTiO_3_ nanospheres, as shown in Fig. 13. Firstly, TiO_2_ sols react with Ba^2+^ ions in a highly alkaline solution to nucleate and form BaTiO_3_ nanoparticles (BT NPs). Then, the BT NPs are loosely aggregated via an orientated attachment mechanism to form single-crystalline-like nanospheres. Finally, the loosely aggregated single-crystalline-like nanospheres are then crystallized during the dynamic hydrothermal process via the reversed crystal growth mechanism. During the reversed crystal growth, the aggregation of nanoparticles may dominate in the early stages of crystal growth, followed by surface crystallization, and then extension from surface to core, to form gapped hollow nanostructures [32, 36, 38, 39]. The formed gapped hollow spheres with thin shells (10–20 nm in thickness) can be destroyed during the long-term stirring to form some small pieces (Fig. 11h). Those small pieces are resolved and then recrystallize on the pores or edges via the Ostwald ripening process, and finally, solid BaTiO_3_ particles are again obtained (Fig. 11i). Actually, the BaTiO_3_ samples obtained under a static hydrothermal process with the same reaction parameters to the dynamic process are composed of solid spherical particles [34]. The continuous stirring seems to favor the surface crystallization and the formation of gapped hollow spheres. It needs to note that different Ti precursors can result in BaTiO_3_ nanocrystals with various morphologies and microstructures even one uses similar hydrothermal process [40].

**Fig. 14:**
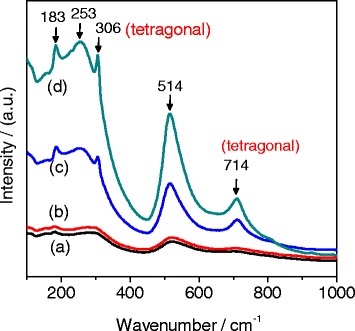
Raman spectra of the BaTiO_3_ samples synthesized with *R*_Ba/Ti_ = 1.2 and [NaOH] = 1.0 mol/L via the dynamic hydrothermal process at 180 °C for various times: **a** 10 min (S18), **b** 1 h (S14), **c** 10 h (S7), and **d** 20 h (S1)

We finally further investigated the phase compositions of the BaTiO_3_ samples using Raman spectroscopy. It is well known that the XRD technique is limited for the crystallographic characterization of fine crystallites owing to extensive broadening of Bragg reflections and generally not very sensitive to transitions involving oxygen displacements. BaTiO_3_ samples usually have a very small tetragonal distortion (*D* = (*c − a*)/*a*) close to 1 %, and therefore, the angular resolution of peak splitting is limited especially for nanocrystal materials. Raman spectroscopy, a kind of vibrational spectroscopy, is sensitive to the abovementioned distortion. We therefore used Raman spectroscopy to characterize the existence and changes in amount of the tetragonal BaTiO_3_ component, for understanding the effect of hydrothermal time on the phase composition of BaTiO_3_ samples obtained with different hydrothermal times. Figure 14 shows the typical Raman spectra of the BaTiO_3_ samples obtained with hydrothermal times of 10 min, 1 h, 10 h, and 20 h. The bands at 183, 253, and 514 cm^−1^ arise from crystalline BaTiO_3_, and they can be observed both in cubic and tetragonal phases, assignable to the scattering from A1(TO) phonons [30]. The tetragonal BaTiO_3_ phase can be determined by checking the relative intensity of the bands at 306 and 714 cm^−1^, which are assigned to the B1 and A1(LO) phonons [30]. What is more, the band at 306 cm^−1^ is assignable to the E(TO) or B1 modes, indicating the asymmetry within the [TiO_6_] octahedra, while the band at 714 cm^−1^ is related to the highest-wavenumber longitudinal optical mode (LO) of A1 symmetry [30]. As Fig. 14 shows, the bands at 306 and 714 cm^−1^ become more and more intense as the hydrothermal time increases from 10 min to 20 h, suggesting that longer hydrothermal time is helpful to enhance the amount of tetragonal BaTiO_3_ phase. The Raman results agree with the XRD patterns (Fig. 10).

## Conclusions

A simple dynamic hydrothermal process has been developed to synthesize hollow and gapped BaTiO_3_ nanospheres with uniform size (93 ± 19 nm) at 180 °C for 10–20 h in aqueous solutions with [NaOH] = 1.0 mol/L and *R*_Ba/Ti_ = 1.2. The effects of hydrothermal temperature and time, NaOH concentration, and Ba/Ti ratio on the morphology and phase of the BaTiO_3_ samples have been investigated systematically on the basis of XRD and SEM results. The optimum conditions for the formation of gapped hollow BaTiO_3_ nanospheres are determined: [NaOH] = 1.0 mol/L and *R*_Ba/Ti_ = 1.2, under dynamically hydrothermal treatments at 180 °C for 10–20 h. The forming paths of the gapped hollow BaTiO_3_ nanospheres can be described as three stages: nucleation (forming nanoparticles), oriented attachment (forming single-crystalline-like spheres), and reversed crystal growth (forming hollow nanospheres with gapped shells). The dynamic hydrothermal process developed here provides an efficient method to obtain highly dispersed BaTiO_3_ nanospheres with gapped hollow structures, and the gapped hollow BaTiO_3_ nanospheres are expected to offer unique performance in dialectical applications.
